# Using spectral-domain optical coherence tomography to evaluate the type and thickness of interdigitation zone band in adult Chinese

**DOI:** 10.1038/s41598-018-30848-1

**Published:** 2018-08-16

**Authors:** Lei Shao, Qing Lin Zhang, Ling Xiao Zhou, Liang Xu, Qi Sheng You, Wen Bin Wei

**Affiliations:** 1 Beijing Tongren Eye Center, Beijing Tongren Hospital, Capital Medical University, Beijing, China; 2 Department of Neurosurgery, Tsinghua University Yuquan Hospital, Beijing, China; 3 Department of Ophthalmology, The First Affiliated Hospital of Xi’an Medical University, Xi’an, Shaanxi China; 4 Beijing Institute of Ophthalmology, Beijing Tongren Hospital, Capital Medical University, Beijing, China

## Abstract

To study types and thickness of interdigitation zone band in adult Chinese subjects, we conducted a cross-sectional study. The population-based Beijing Eye Study 2011 included 3468 individuals with a mean age of 64.6 ± 9.8 years. 263 people (263eyes) with a mean age of 64.8 years were randomly selected cases without macular diseases included in the study. A detailed ophthalmic examination was performed including SD-OCT for measurement of the thickness of interdigitation zone band. There are two types of interdigitation zone band; the type1 which can distinguish RPE–BM complex in 170 eyes; and the Type 2 which the two layers merged involved 93 eyes. In type1, the mean thickness of the interdigitation zone band was significantly thicker in the foveal center (16.46 ± 2.92 μm), then nasal macular region (16.19 ± 2.69 μm), temporal macular region (15.73 ± 2.68 . μm), superior region (15.72 ± 2.70 μm), and inferior macular region (14.84 ± 2.63 μm) (*P* all < 0.05). And the mean thickness of the interdigitation zone band in the foveal center associated with the subfoveal choroidal thickness (*P* = 0.025) and level of education (*P* = 0.033). The increase in the thickness of the interdigitation zone band may play a role in the pathophysiologic features of various age-related ocular conditions.

## Introduction

Interdigitation zones was defined as the interdigitation between the cone outer segments and the RPE microvilli corresponded to the ensheathment of the cone outer segments by the processes of the RPE in a structure known as the contact cylinder, whose function was not clear as that of the RPE-Bruch’s membrane complex. While the retinal pigment epithelium (RPE) which connected with the interdigitation zones performs a variety of functions in vectorial transport, supporting the functions of photoreceptors and other cells in the neural retina, forming the outer blood-retinal barrier and so on^1–3^. Recently, with better resolution, spectral domain optical coherence tomography (SD-OCT) has been used to examine the foveal microstructures in greater detail^4–6^. It was the landmark study by Staurenghi G *et al*.^7^ developed a consensus nomenclature for the classification of retinal and choroidal layers and demonstrated that two highly reflective bands can be seen in the outer retina in the SD-OCT images, which were representative of the interdigitation zones and the RPE-Bruch’s membrane complex. Although the functions of interdigitation zones were not clear, studies revealed that destructions of these microstructures can be indicated in different retinal diseases, including retinal detachment^8^, age-related macular degeneration^3^, foveomacular vitelliform dystrophy^9^, central serous chorioretinopathy^10^, and acute, posterior multifocal placoid pigment epitheliopathy^11^. The primary aim of our research is to study the type and thickness of interdigitation zone band in adult Chinese subjects by SD OCT and its correlation with systemic and ocular biometric parameters with a relatively large study population.

## Methods

Our study based on the Beijing Eye Study 2011, which is a population-based cross-sectional study in Northern China. The study was carried out in 5 communities in the urban district and in 3 communities in the village district of Beijing. People over 50 years of age are included into the study. The 8 communities had a total population of 4403 individuals conforming to the inclusion criteria, 3468 individuals (1963 (56.6%) women) participated in the eye examination, corresponding to an overall response rate of 78.8%. The mean age of all participants were 64.6 ± 9.8 years (median, 64 years; range, 50–93 years). The rural part included 1633 (47.1%) subjects (943 (57.7%) women) and the urban part included 1835 (52.9%) subjects (1020 (55.6%) women). All examinations were carried out in the communities, either in schoolhouses or in community houses. The participants were asked to provid information on the general situation and received physical and ophthalmic examination. The study has been described in detail^12^.

According to the Declaration of Helsinki, the Medical Ethics Committee of the Beijing Tongren Hospital approved the study protocol and all participants gave informed written consent, and all experiments were performed in accordance with relevant guidelines and regulations.

263 people with apparently normal eyes were randomly selected cases, but with different age and axial length from Beijing eye study 2011 database. The inclusion criterion was the participants without eye disease or eye surgery, which were found by ophthalmic examination and questionnaire. The macular region was imaged by spectral-domain optical coherence tomography (SD-OCT; Spectralis®, Wavelength: 870 nm; Heidelberg Engineering Co., Heidelberg, Germany). The examination and the methods of measurement had been described in detail^13^. Only the right eye of each study participant was assessed.

**Figure 1:**
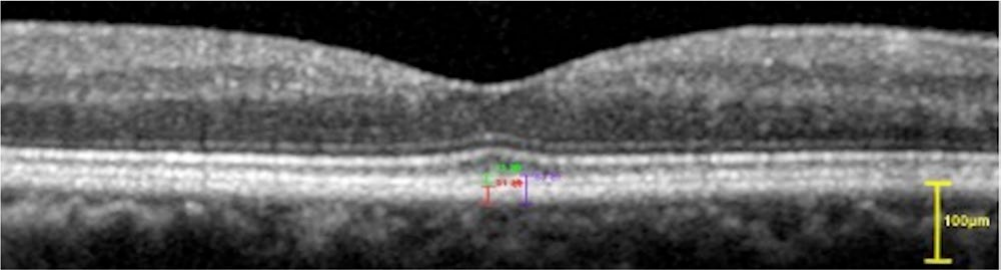
Optical coherence tomogram of the RPE–BM complex and the interdigitation zone band (Type 1). The inner and outer borders of the RPE–BM complex and the interdigitation zone band were manually segmented. The thickness of the two layers were measured separately at first; the red line showed the subfoveal thickness of RPE–BM complex; the green line showed the subfoveal thickness of the the interdigitation zone band. Then measured the two layers together (from the inner border of interdigitation zone band to the outer border of the RPE–BM complex) and showed with the purple line.

**Figure 2:**
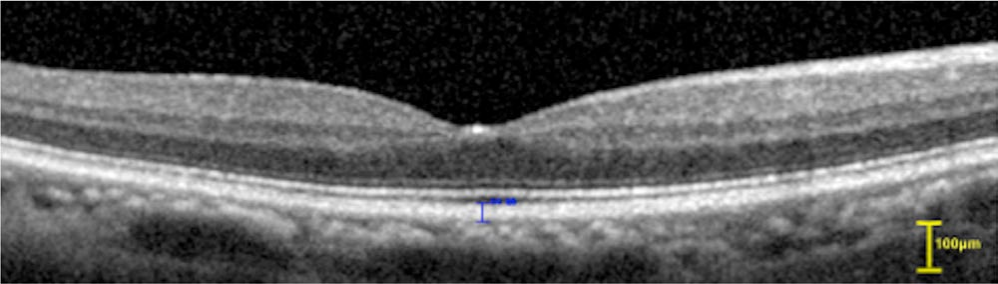
Optical coherence tomogram of the RPE–BM complex and the interdigitation zone band (Type 2). The two layers appear to have merged (without the dark band between the two layers) on the OCT image, and both were measured together with the blue line.

There are two types of interdigitation zone band; the type1 which can be distinguished with RPE–BM complex (Fig. 1); and the Type 2 which merged with RPE–BM complex (Fig. 2). For type 1: the thickness of the interdigitation zone band and RPE–BM complex was measured separately at first; then measured the two layers together (from the inner border of interdigitation zone band to the outer border of the RPE–BM complex). For type 2: the thickness of the interdigitation zone band and RPE–BM complex should be measured together only. The inner and outer borders of the RPE–BM complex and the interdigitation zone band were manually segmented (Fig. 1). Each image was measured at 5 locations: in the fovea, and in the outer extreme section superior to the fovea, inferior to the fovea, temporal of the fovea and 2 mm nasal to the fovea in direction to the optic disc. The images were taken by one technician (CXC) and the images were assessed by two ophthalmologists (LS, KFD).

Statistical analysis was performed by using SPSS software package (SPSS for Windows, version 20.0, SPSS, Chicago, IL, USA). In the first step, differences for the thickness of RPE–BM complex and the interdigitation zone band measured together in four quadrants and fovea were compared between the type 1 and type 2. In the second step, we performed a univariate linear regression analysis with the thickness of interdigitation zone band of type 1 as a dependent parameter and ocular and general parameters as independent parameters. In the third step, we performed a multivariate linear regression analysis, with the thickness of interdigitation zone band of type 1 as a dependent parameter and all those parameters as independent parameters that were significantly associated with it in univariate analysis. The thickness of RPE–BM complex and the interdigitation band were described by the mean values (presented as mean ± standard deviation). Categorical variables were assessed individually with the chi-square test, and the Fisher exact test was used for samples with an expectancy of less than 5. Continued data were analyzed using an independent sampled t-test. The paired t test was used to analyze differences in thickness by location in the macula. Simple liner regression was calculated for variations in the thickness of the interdigitation zone band relative to systemic and ocular risk factors. Multiple linear regression was used to evaluate the explanatory variables with regard to the dependent variable. 95% Confidence intervals (CI) were presented. All P-values were 2-sided and were considered statistically significant when the values were less than 0.05.

## Results

263 people (263eyes) were included in the study. 117 (44.5%) subjects were male. The mean age was 64.8 ± 9.6 years (median: 65 years; range: 50 to 89 years), mean refractive error (spherical equivalent) was −0.12 ± 1.99 diopters (median: 0.25 diopters; range: −5.75 to +7.00 diopters), and mean axial length was 23.18 ± 1.08 mm (median: 23.13 mm; range: 19.94–26.47 mm).

The Type1 group included 170 cases (81(47.6%) male)); and the Type 2 group involved 93 eyes (36(38.7%) male). The Type 2 group, as compared with the Type1 group, was significantly (*P* < 0.001) older (68.7 ± 9.3 years versus 62.7 ± 9.0 years) and did not vary significantly in gender (*P* = 0.16), axial length (*P* = 0.96) and refractive error (*P* = 0.28).

**Figure 3:**
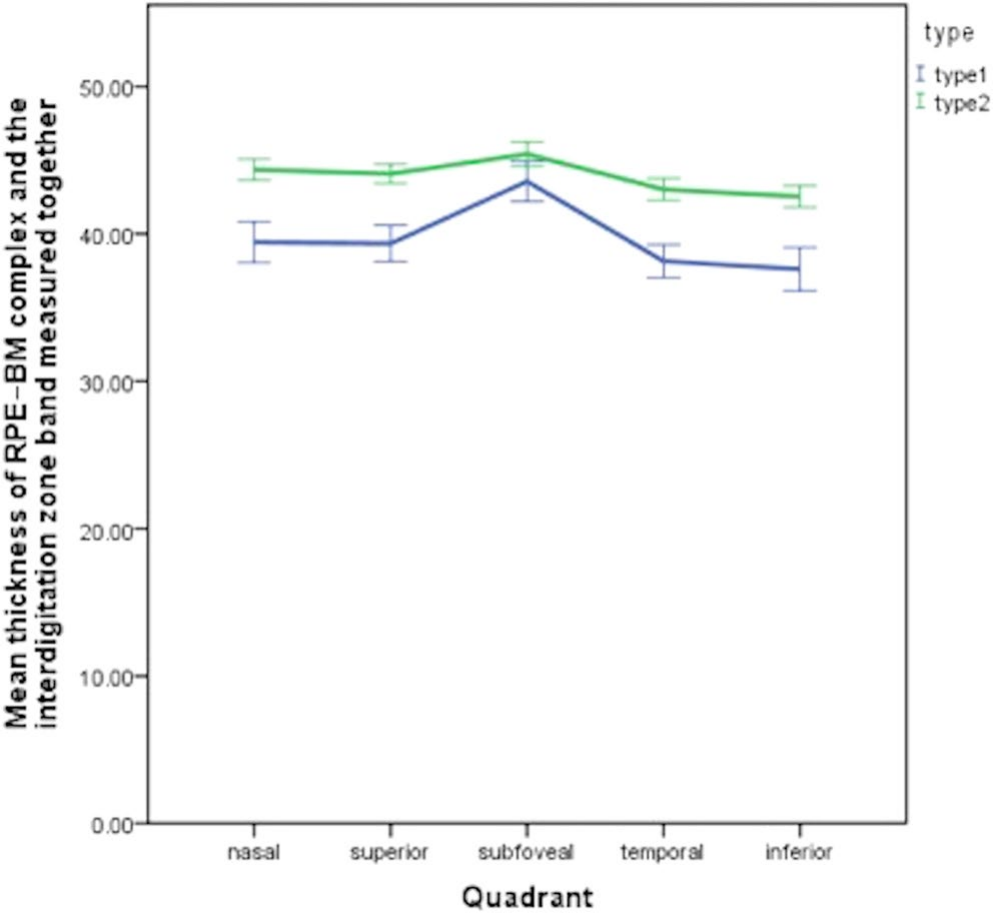
Average thickness of RPE–BM complex and the interdigitation zone band measured together and 95% confidence Interval (CI) at different locations (error bar shows the 95%CI).

Table 1 and Fig. 3 compares the mean thickness of RPE–BM complex and the interdigitation zone band measured together in both groups at all locations. There was a similar trend, with the inferior quadrant exhibiting the thinnest thickness of RPE–BM complex and the interdigitation zone band among all four quadrants in both groups; then were the temporal, superior and nasal quadrant successively; and the subfoveal thickness was the thickest. At each quadrant, the mean thickness of the Type 2 group was thinner than that of the Type1 group (*P* all < 0.05).

**Table 1:** Average thickness of RPE–BM complex and the interdigitation zone band measured together and 95% confidence Interval (CI) at different locations with line scans.

Sector	Group Type 1		Group Type 2		Mean difference, μm	95%CI, μm		*P*
	Mean	SD, μm	Mean	SD, μm		Lower bound	Upper bound	
Subfoveal	45.42	5.40	43.56	6.64	−1.86	−3.45	−0.27	0.022
Superior	44.07	4.32	39.34	5.98	−4.73	−5.99	−3.46	<0.001
Inferior	42.53	4.81	37.60	7.12	−6.01	−6.56	−3.30	<0.001
Temporal	43.01	4.90	38.14	5.46	−4.87	−6.17	−3.58	<0.001
Nasal	44.36	4.76	39.43	6.71	−4.93	−6.49	−3.38	<0.001

Two layers were measured together (from the inner border of interdigitation zone band to the outer border of the RPE–BM complex).

For the Type1 group, the thickness of RPE–BM complex and the interdigitation zone band were measured separately. In total 170 cases, the mean age was 62.7 ± 9.0 years (median: 62 years; range: 50 to 87 years), mean refractive error (spherical equivalent) was −0.02 ± 1.86 diopters (median: 0.25 diopters; range: −5.38 to + 7.00 diopters), and mean axial length was 23.18 ± 1.06 mm (median: 23.12 mm; range: 19.94–26.47 mm).

The mean thickness of the RPE–BM complex at the foveal center was significantly (P < 0.001) thicker (25.09 ± 3.98 μm; range: 17–37 μm) than that at 2.0 mm distant from nasally (23.65 ± 3.64 μm; range: 13–36 μm) and superiorly (23.34 ± 3.29 μm; range: 15–33 μm), where it was significantly (P < 0.001) thicker than inferiorly (22.48 ± 3.29 μm; range: 14–34 μm) and temporally (22.44 ± 3.47 μm; range: 15–32 μm).

The mean thickness of the interdigitation zone band was significantly (P = 0.003) thicker in the foveal center (16.46 ± 2.92 μm; range: 10–27 μm) and in the nasal macular region (16.19 ± 2.69 μm; range: 10–26 μm) than in the temporal macular region (15.73 ± 2.68 . μm; range: 8–24 μm) and superior region (15.72 ± 2.70 μm; range: 6–25 μm), in which it was significantly (P < 0.001) thicker than in the inferior macular region (14.84 ± 2.63 μm; range: 8–22 μm).

In univariate analysis, the foveal thickness of the interdigitation zone band was significantly associated with younger age (*P* = 0.050), level of education (*P* = 0.046), subfoveal choroidal thickness (*P* = 0.024); and marginal related to history of absence of hyperlipidemia (*P* = 0.088). (Table 2) It was not significantly (all *P* > 0.05) associated with the systemic parameters of gender, body height, weight, rural region of habitation, systolic and diastolic blood pressure, serum concentrations of glucose, high-density lipoproteins, low-density lipoproteins, cholesterol and triglycerides, presence of diabetes mellitus, smoking and alcohol consumption, aspirin intake and frequency of reported snoring, hypertension history; with the ocular parameters of best corrected visual acuity, axial length, refractive error, subfoveal retinal thickness, anterior chamber depth, lens thickness, ocular perfusion pressure, corneal thickness, curvature and diameter, and pupil diameter (Table 2).

**Table 2:** Univariate associations between foveal thickness of the interdigitation zone band and ocular and general parameters.

Parameter	Unstandardized Coefficients (B)	95% Confidence Interval	Standardized Coefficients (beta)	P-Value
**Systemic Parameters**				
Age (years)	−0.05	−0.10, 0.00	−0.15	0.050
Gender	−0.34	−1.23, 0.55	−0.06	0.452
Body Height (cm)	0.03	−0.02, 0.09	0.10	0.219
Body Weight (kg)	0.00	−0.03, 0.04	0.15	0.844
Rural/Urban Region	0.17	−0.72, 1.07	0.03	0.700
Level of Education	0.52	0.01, 1.03	0.16	0.046
Systolic Blood Pressure (mmHg)	0.01	−0.01, 0.03	0.06	0.437
Diastolic Blood Pressure (mmHg)	0.02	−0.02, 0.05	0.07	0.393
High-Density Lipoproteins(mmol/l)	−0.14	−1.35, 1.07	−0.02	0.823
Low-Density Lipoproteins(mmol/l)	−0.03	−0.61, 0.56	−0.01	0.931
Cholesterol (mmol/l)	−0.06	−0.60, 0.48	−0.02	0.826
creatinine (mmol/l)	0.00	−0.04, 0.04	0.00	0.994
Triglycerides (mmol/l)	−0.00	−0.15, 0.14	−0.00	0.962
Glucose (mmol/l)	0.11	−0.33, 0.55	0.05	0.611
Diabetes Mellitus	0.40	−1.19, 1.98	0.04	0.621
Smoking	−0.35	−0.90, 0.20	−0.10	0.209
Alcohol Consumption	0.03	−0.20, 0.26	0.02	0.815
Aspirin Intake	0.66	−0.31, 1.62	0.12	0.179
Snoring	−0.34	−0.98, 0.29	−0.09	0.284
Hypertension history	−0.71	−1.60, 0.17	−0.13	0.114
Hyperlipidemia history	−0.11	−0.23, 0.02	−0.13	0.088
**Ocular Parameters**				
Refractive Error (D)	0.04	−0.20, 0.28	0.03	0.747
Best Corr. Visual Acuity (logMAR)	1.62	−0.69, 3.94	0.12	0.168
Axial Length (mm)	−0.18	−0.61, 0.26	−0.06	0.419
Centre Corneal Thickness (µm)	0.00	−0.01, 0.01	0.00	0.958
Anterior Chamber Depth (mm)	0.32	−0.94, 1.58	0.04	0.620
Lens Thickness (mm)	−0.40	−1.81, 1.01	−0.05	0.578
Corneal Curvature (mm)	−0.62	−2.39, 1.14	−0.06	0.489
Corneal Diameter (mm)	0.16	−0.34, 0.66	−0.05	0.530
Pupil Diameter (mm)	−0.01	−0.60, 0.59	−0.00	0.985
Ocular Perfusion Pressure (mmHg)	0.02	−0.13, 0.16	0.02	0.828
Subfoveal choroidal thickness (µm)	0.01	0.00, 0.01	0.17	0.024
Subfoveal retinal thickness(µm)	0.00	−0.03, 0.03	0.02	0.852

P-values were less than 0.05.

The multiple linear regression analysis (R^2^ = 0.274, condition index = 5.91) showed that subfoveal choroidal thickness (*P* = 0.025; beta: 0.17; B: 0.01 (95%CI: 0.00, 0.01) VIF = 1.12) and level of education (*P* = 0.033; beta: 0.16; B: 0.55 (95%CI: 0.05,1.06) VIF = 1.11) was also significantly related to the subfoveal thickness of interdigitation zone band. But age (*P* = 0.269 VIF = 1.14) and history of hyperlipidemia (*P* = 0.234 VIF = 1.12) were not significantly (P all > 0.05) correlated with it (Table 3).

**Table 3:** Multivariate associations between foveal thickness of the interdigitation zone band and ocular and general parameters.

Parameter	*P*-Value	Standardized Coefficient beta	Regression Coefficient	95% Confidence Intervals	Variance Inflation Factor
subfoveal choroidal thickness(μm)	0.025	0.17	0.01	0.00, 0.01	1.12
level of education	0.033	0.16	0.55	0.05, 1.06	1.11

R^2^ = 0.274, condition index = 5.91.

## Discussion

In our population-based study with a relatively large study population, we found that the interdigitation zone band have two different types of the structures showed by the OCT; the interdigitation zone band and the RPE–BM complex in the type1 can be distinguished, but which in the Type 2 was merged. The present research also reveals that the cases of Type 2 were older than the type1. The differences of the thickness of the individual RPE–BM layers with age observed in the present study are mostly in concordance with previous studies^14–16^, and confirmed in a histomorphometric study by Bonilha VL^17^, which demonstrated that several structural changes occur as the aging RPE, including loss of melanin granules, increase in the density of residual bodies and accumulation of lipofuscin, accumulation of basal deposits on or within Bruch’s membrane, formation of drusen, and thickening of Bruch’s membrane.

In present study, we also measured thickness of the interdigitation zone band in Type1 group, only this type of the interdigitation zone can be distinguish with RPE–BM complex. The results revealed that mean thickness was thickest in the foveal center (16.46 μm) and in the nasal region (16.19 μm), followed by in the temporal region (15.73 μm) and superior region (15.72 μm), and finally in the inferior section (14.84 μm). At the same time, the multiple linear regression analysis showed that subfoveal choroidal thickness (*P* = 0.025; beta: 0.17) and level of education (*P* = 0.033; beta: 0.16) was significantly related to the subfoveal thickness of interdigitation zone band.

These results was hard to be compared with other investigations, since only few research applied OCT technology for imaging of the interdigitation zone band, and even measured the thickness of the band. Staurenghi G and *et al*.^7^ re-denominate the hyperreflective band above the RPE-BM complex in OCT images as interdigitation zone band recently, instead of the previous name of Verhoeff’s membrane. Verhoeff’s membrane has been described as the tight junctional complexes between RPE cells that are visible as a band on electron microscopy. While the hyperreflective zone lies anterior to the RPE complex and thus has been proposed to represent photoreceptor outer segment tips as its anatomic correlate^6,18^. The present study also showed the band has a thickness (16.46 μm) greater than would be suggested by just the outer segment tips and may represent the interdigitation of the apical processes of the RPE with the cone outer segments. As such, stating the reflection is solely from the cone tips. The possible reasons for the differences of the quadrantal distribution in the thickness of the interdigitation zone band complex may be that velocity of the loss of cells and the accumulation of metabolic stains would be different.

In our study, the subfoveal choroidal thickness (*P* = 0.025; beta: 0.17) and level of education (*P* = 0.033; beta: 0.16) was significantly related to the subfoveal thickness of interdigitation zone band. Although there was few study to report the related factors of the interdigitation zone band; clinically, the finding of our study concerning the association between the subfoveal thickness of interdigitation zone band and thicker subfoveal choroidal thickness may be of interest since a thick SFCT has been reported to be associated with increased best corrected visual acuity after adjusting for confounding factors such as age, refractive error and ocular diseases^19^. Future studies may address whether a thin subfoveal choroidal thickness in some eyes with destructive interdigitation zone band as compared to eyes with undamaged interdigitation structures is associated with a lower best corrected visual acuity in some retinal disease or after therapy.

Potential limitations of our study should be mentioned. First, a major concern in any prevalence study is nonparticipation. The Beijing Eye Study 2011 had a reasonable response rate of 78.8%, however, differences between participants and non-participants could have led to a selection artifact. Second, the present study is the relatively small sample size which randomly selected cases from epidemiologic research, and the fact that it was based on cross-sectional data rather than longitudinal data. Third, thickness of interdigitation zone band was examined only in the right eye of each study participant, so that inter-eye differences and their associations with inter-eye differences of other parameters could not be assessed. Fourth, as in any population-based study, our investigation included all eligible and participating subjects from the study region; thus, patients with diseases, such as disorders of the optic nerve and macula, and these diseases may have affected the thickness of interdigitation zone band; besides, some measurement was close to the spatial resolution limit of the OCT, manual measurement of thickness might be a source of error in results analysis. Future studies may address whether related diseases were associated with abnormalities of interdigitation zone band. Besides OCT, photoacoustic microscopy (PAM) can achieve multilayered histology-like imaging of the tissue surface, it may be another potential tool for future eye study^20,21^.

In conclusion, the interdigitation zone band can be seen in two types; the type1 which can be distinguish with RPE–BM complex; and the Type 2 which merged with RPE–BM complex, the mean thickness of the interdigitation zone band in the foveal center was 16.46 ± 2.92 μm in elderly subjects with a mean age of 63 years. And it associated with the subfoveal choroidal thickness and level of education.
